# A Case Report of Salvage Radiotherapy for a Patient with Recurrent Gastric Cancer and Multiple Comorbidities Using Real-time MRI-guided Adaptive Treatment System

**DOI:** 10.7759/cureus.2471

**Published:** 2018-04-12

**Authors:** Seok-Joo Chun, Seung Hyuck Jeon, Eui Kyu Chie

**Affiliations:** 1 Department of Radiation Oncology, Seoul National University Hospital

**Keywords:** mri-guided, respiratory gating, adaptive planning, radiotherapy

## Abstract

The stomach is one of the most deforming organs caused by respiratory motions and daily variation by food intake. Applying radiotherapy has been quite a challenge due to the high risk of missing the target as well as radiation exposure to large volumes of normal tissue. However, real-time magnetic resonance (MR)-guided radiotherapy with adaptive planning could focus the high dose radiation to the target area while minimizing neighboring normal tissue exposure and compensate for not only daily but real-time variation. Here is a case report of a patient with recurrent gastric cancer and multiple co-morbidities, unsuitable for both resection and chemotherapy, who underwent MR guided adaptive radiotherapy.

## Introduction

Gastric cancer is one of the most common cancers worldwide. More than 30,000 patients have been diagnosed and roughly one-fifth of the patients eventually die annually in South Korea [1]. Despite the standard of care being composed of surgery and perioperative adjuvant treatment, many patients confront relapse where the standard of care has not been settled. Salvage operation for resectable lesions is recommended by the National Comprehensive Cancer Network (NCCN) guideline, while other guidelines recommend salvage chemotherapy based on a trial which demonstrated the clinical benefits of palliative chemotherapy over best supportive care [2-3].

Despite the high incidence and prevalence of isolated loco-regional recurrence, radiotherapy is not often used in Korea [1]. Many reasons contribute to this low utilization. First, radiation failed to show benefit over chemotherapy in the prospective randomized trial (ARTIST trial) and in the large-scale cohort analysis conducted by the Dutch Gastric Cancer Group after D2 lymph node dissection, which is the current standard of care [2-3]. Secondly, there are frequent relocations and substantial daily volume variation of the stomach caused by respiratory motion and daily food intake. To compensate for this uncertainty, radiotherapy is delivered to a rather large clinical target volume (CTV) and even larger planning target volume (PTV). This would have led to increased risk of treatment-related morbidity, which may have offset the clinical benefit of radiotherapy. However, innovative technologies have awakened new possibilities. By using real-time magnetic resonance imaging (MRI) guidance and daily adaptation, radiotherapy can be delivered to a highly focused target with minimal exposure to neighboring normal tissues. Here, we present the case report of a patient with recurrent stomach cancer and multiple co-morbidities, unfit for both resection and chemotherapy, who was able to undergo salvage radiotherapy utilizing the high-end radiotherapy delivery technique.

## Case presentation

**Figure 1:**
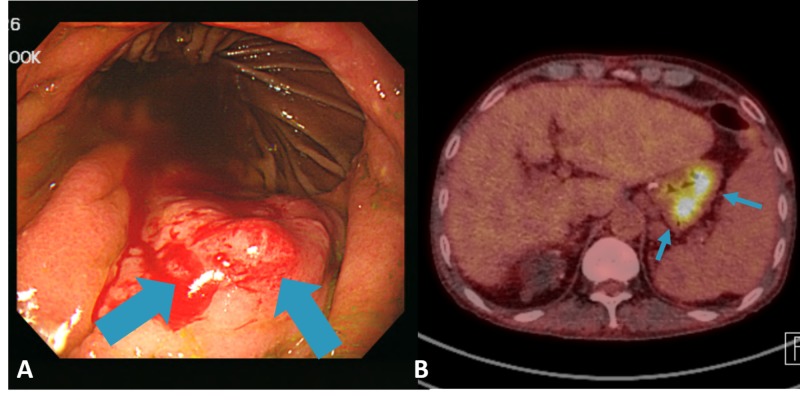
(A) Nodular mass in greater curvature with bleeding on esophagogastroduodenoscopy and (B) diffuse hypermetabolism along remnant stomach with combined inflammation in the FDG-PET CT FDG-PET: fluorodeoxyglucose positron emission tomography; CT: computed tomography.

A 52-year-old Korean male underwent distal gastrectomy for stomach cancer in 1999. Pathology report revealed early gastric cancer. Adjuvant treatment was not offered to the patient. The patient had multiple serious comorbidities including end-stage renal disease, for which patient was on hemodialysis and liver cirrhosis, which was associated with underlying viral hepatitis. The patient underwent regular surveillance with esophagogastroduodenoscopy (EGD) biannually. The patient was also under surveillance with serum alpha fetoprotein, liver function panel, and either ultrasonography or computed tomography (CT) scan of the liver following the Korean national guideline for high-risk patients with hepatocellular carcinoma. There were no signs of recurrence as of November 2016 when the patient had both EGD and CT. On July 2017, the patient visited the emergency room due to a sudden onset of melena. Emergency EGD revealed a 2 cm sized nodular mass in the greater curvature with bleeding (Figure 1A). Biopsy report showed moderately differentiated adenocarcinoma, identical to initial pathologic diagnosis. Stomach CT revealed a 2-cm sized cancer in the remnant stomach. Primary lesion on CT was suspicious for muscularis propria invasion. However, there was no evidence of lymph node or distant metastasis. Fluorodeoxyglucose positron emission tomography (FDG-PET) showed diffuse hypermetabolism in the stomach without evidence of metastasis elsewhere (Figure 1B).

Remnant total gastrectomy was planned for this clinically staged T2N0 recurrent stomach cancer. On open laparotomy, however, severe adhesion was found. Due to high risk of incomplete resection and bleeding risk due to multiple comorbidities, further surgical exploration was not sought. The attending medical oncologist recommended against palliative chemotherapy. The underlying rationale was that the risk of adverse events from the anticipated treatment, including fatal hepatic failure, outweighed the survival benefit. After multidisciplinary clinic discussion, radiotherapy was offered as the only viable option besides best supportive care.

**Figure 2:**
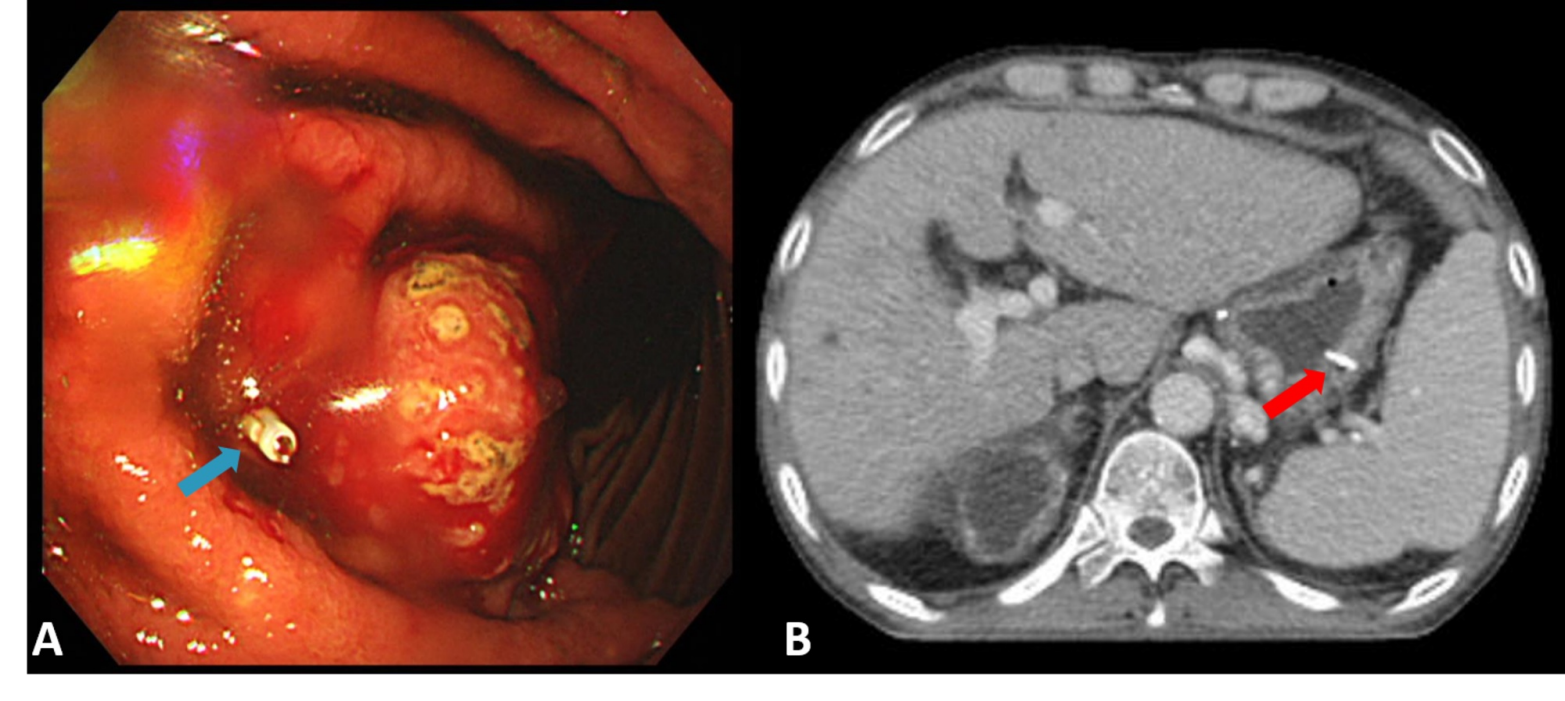
(A) Metal clip was placed via EGD proximal to the gastric tumor (blue arrow) and (B) metal clip placed in greater curvature of the stomach on simulation CT (red arrow) EGD: esophagogastroduodenoscopy; CT: computed tomography.

A metal clip was placed via EGD to allocate and later monitor tumor motion prior to CT and MR simulation (Figures 2A-2B).

**Figure 3:**
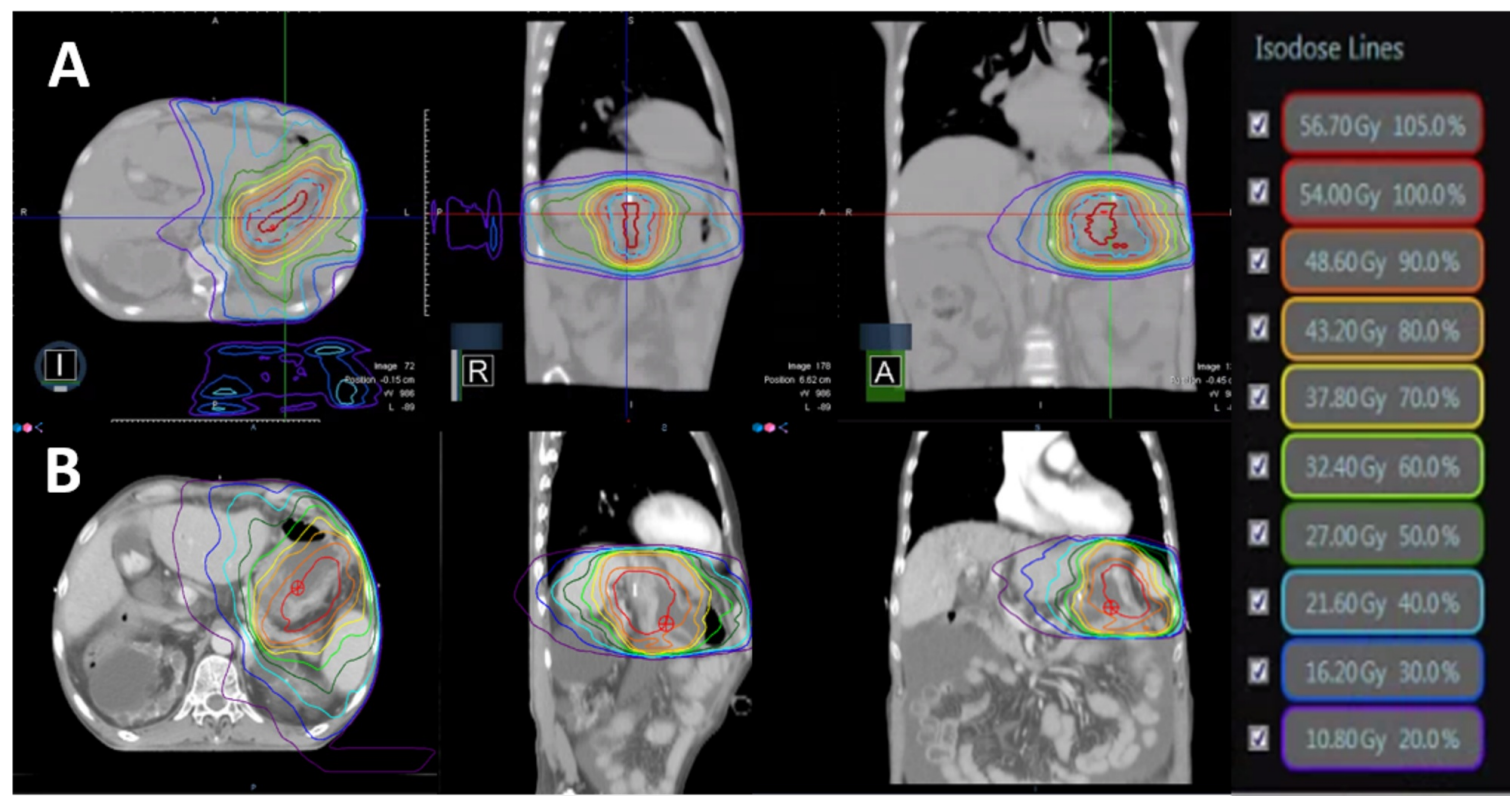
The representative dose distributions of (A) MRIDian tri-cobalt-60 system and (B) Linac

The patient underwent CT simulation with pneumatic compression to minimize the stomach motion. The patient also underwent MR simulation on 0.35-T MRI-guided radiotherapy system (MRIdian® System, ViewRayTM Inc, Oakwood Village, Ohio, US) with surface coils on the abdomen. The same position CT was also obtained for treatment planning. All simulation procedures were done in a single day. Simulation images were compared to diagnostic images of CT and PET. Lesion on greater curvature with contrast enhancement distal to metal clip on simulation CT was defined as gross tumor volume (GTV). PTV was defined as 0.8 cm margin expansion from GTV, where 54.0 Gy in 27 fractions was prescribed. Intensity-modulated radiotherapy (IMRT) plans were independently constructed for TrueBeam® STX (Varian Medical Systems Inc., Palo Alto, California, US), 10MV FFF beam with EclipseTM Treatment Planning System (Varian Associates, Palo Alto, California, US) and MRIdian tri-cobalt-60 system (ViewRay Inc., Oakwood Village, Ohio, US). A volumetric arc was chosen for the linac plan, whereas fixed beam arrangement was used for tri-60-cobalt plan. For the MR-guided plan, the presence of transverse magnetic fields was taken into account during optimization and calculation phase. Generated plans were compared for dose distributions of GTV, PTV, and various neighboring organ at risks (OAR’s) including remnant stomach, duodenum, both kidneys, liver, and spinal cord. GTV volume was smaller for MR plan, where treatment was gated compared to the linac plan; compressed ITV was used as the target. A comparison of other plan parameters can be seen in Table 1 and Figure 3.

**Table 1:** Dose profile comparison between Linac plan and Tri-co-60 plan GTV: gross tumor volume; PTV: planning target volume; D_max_: maximum point dose of target; D_min_: minimum point dose of target; D_30%_: maximum dose of 30% of target; D_30cc_: maximum dose of 30cc of target; D_3cc_: maximum dose of 3cc of target; D_95%_: maximum dose of 95% of target. * Volume of PTV_54_

	Linac	Tri-co-60
Stomach		
D_max_	56.5	57.3
D_30%_	53.8	52.3
D_30cc_	54.9	56.3
D_3cc_	55.6	56.6
GTV		
Volume (cc)	26.8	23.2
D_min_	52.6	53.1
D_95%_	54.3	54.1
PTV		
Volume (cc)	102.4^*^	126.6
D_min_	49.1	51.8
D_95%_	54.0	54.0

**Figure 4:**
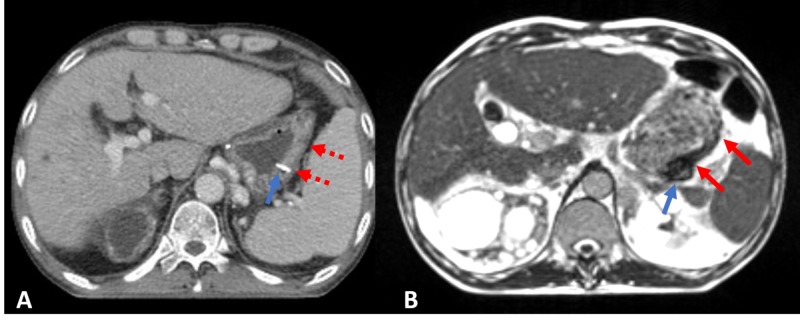
(A) Clip (blue arrow) and stomach cancer lesion (red arrow) on simulation CT with contrast and (B) clip (blue arrow) and stomach cancer lesion (red arrow) on simulation MRI Notice that lesion extent is much more marked on MR compared to CT. MRI: magnetic resonance imaging; CT: computed tomography.

Of note, change in air cavity for daily variation was not taken into account for both plans. Information on daily changes was not available for the linac plan. Though MR was taken daily, dose calculation was based on a deformed modification of simulation CT. Tri-60-cobalt plan was chosen over the linac plan for several reasons such as 1. better visualization of the target and OAR using MR, 2. respiratory gating technology to compensate for respiratory motion, 3. capability of adaptation to variation of daily gastric volume, and 4. lower stomach 30% dose and scattered hot spots (Figure 4).

**Figure 5:**
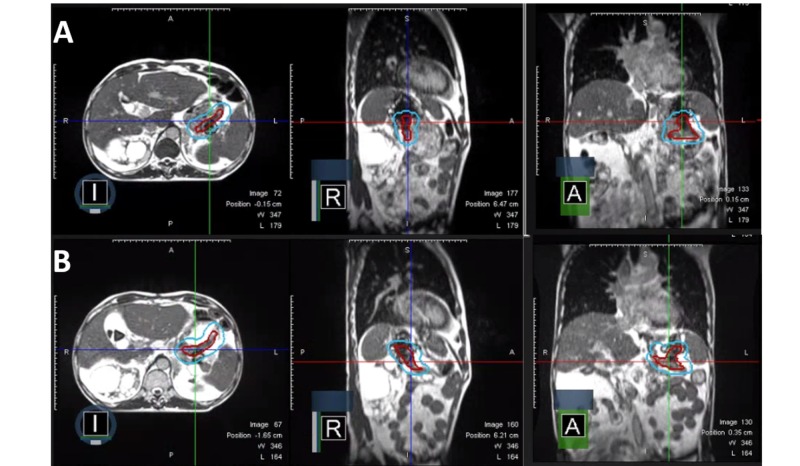
Axial, sagittal, and coronal view of (A) the plan on simulation and (B) of the adaptive plan on the first treatment day

**Figure 6:**
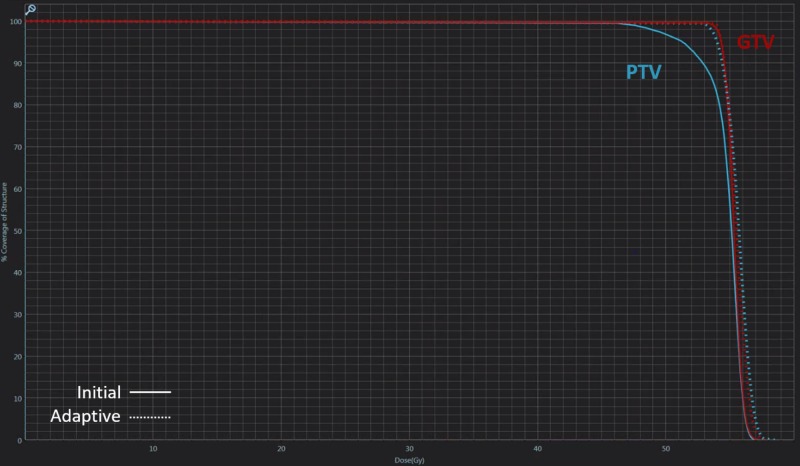
Comparison of dose-volume histogram for the initial plan (straight line) and adaptive plan (dotted line) Notice the marked difference in planning target volume coverage.

The patient was treated with real-time MRI guided tri-cobalt-60 delivery system via deformable image registration-based beam control in the sagittal plane at four frames per second. At each treatment, volumetric MRI in the treatment position was taken with breath holding and the image was registered by the attending radiation oncologist and/or radiation technologists. On the first day of treatment, acquired MR showed huge discordance compared to the initial simulation MR (Figure 5). Target volumes and OAR were re-contoured and the plan was re-optimized. Compared to the initial plan, the adaptive plan had better coverage of the target without significant difference in OARs thus confirming the quality assurance (QA) procedure of the adaptive plan followed (Figure 6). The patient was treated with the adaptive plan for the remaining fractions without a major deviation on the first day of the treatment. The patient experienced only grade 1 nausea throughout the treatment sessions.

**Figure 7:**
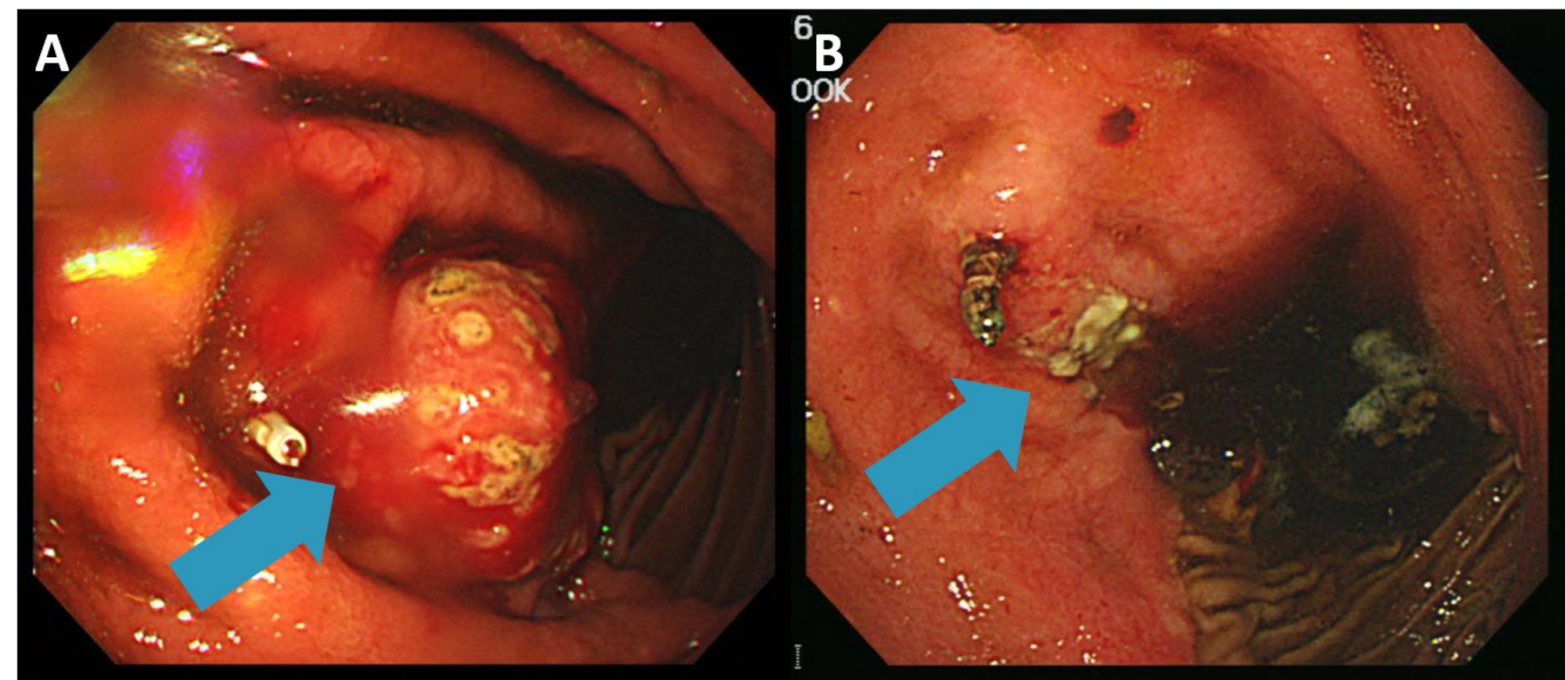
(A) Bulging mass with bleeding distal to the metal clip on pre-treatment EGD and (B) regressed mass with ulceration distal to clip on follow-up EGD EGD: esophagogastroduodenoscopy

After six weeks of completion of radiotherapy, the patient re-visited the emergency room for recurrent melena. Follow-up EGD showed regressed mass with ulceration and minute tumor bleeding, which was conservatively controlled (Figure 7). The patient will be regularly followed to monitor treatment response, as well as disease and/or treatment-related adverse events.

## Discussion

There has been a continuous debate on the role of radiotherapy in patients with stomach cancer. Radiotherapy in combination with chemotherapy showed a survival benefit as compared to surgery alone in a postoperative setting in a landmark Intergroup 0116 trial, where the positive impact was sustained after extended follow-up [2-3]. However, this trial has been criticized for having non-standard surgery as a backbone. There have been suggestions that the role of chemoradiotherapy was to supplement insufficient surgery. Three prospective trials, namely MOSAIC, ACTS-GC, and CLASSIC, incorporating perioperative chemotherapy have confirmed the role of chemotherapy in stomach cancer [2-3]. Meanwhile, the additional role of chemoradiotherapy over chemotherapy have been questioned. Small prospective studies have failed to demonstrate a statistically significant gain of chemoradiotherapy over chemotherapy alone [1-3]. The Dutch Gastric Cancer Group conducted a prospective cohort based large-scale retrospective analysis. In this analysis, it was suggested that chemoradiotherapy offers locoregional control benefit over chemotherapy, but not the overall survival. Furthermore, in a subgroup analysis, this gain in local control was only evident in a patient who underwent D1 lymph node dissection [2-3]. Although disease-free survival gain in patients with nodal involvement was suggested in further analysis and following meta-analysis [4], the primary end point was not met in the ARTIST trial. Although the anticipated TOPGEAR trial has just recently finished patient accrual and ARTIST-II is underway, the more recently reported CRITICS trial also failed to demonstrate the benefit of adding radiotherapy to chemotherapy over chemotherapy alone [1]. There have been several anecdotal reports on the role of radiotherapy in a palliative setting. Effect of radiotherapy is quite evident for bleeding control. However, it is less clear on pain or obstruction [5]. Role of radiotherapy in salvage setting beyond symptom palliation is even vaguer with a very limited number of reports if any.

In our institution, radiotherapy was seldom administered owing to the previously stated reasons [6]. Deformation of the stomach has been reported to be quite considerable with a maximum overlap of volume ranging from 30% to 95% [7]. Gastric motions caused by respiration were reported as high as 11 mm [8]. In the past, more than 1 cm margin was recommended from CTV to PTV for conventional radiotherapy in prospective trials [2].

To overcome interfractional gastric motion, techniques such as image guidance, respiratory gating, and adaptive plan in various combinations have been developed to better identify GTV and decrease doses to OAR’s. In pancreatic cancer, daily image guidance and the adaptive delivery system showed better target coverage and dose distribution to duodenum which could, in turn, reduce fatal toxicities [7]. This combined effort of technical innovations opened new possibilities in treating stomach cancer as well, where motion and deformation are much more marked. In the past, an adaptive plan meant repeated CT simulation followed by re-plan and then QA, spanning over days, if not weeks. But with the help of innovative treatment-planning and delivery system, it takes less than 30 minutes for re-contouring, re-planning, and re-optimization, if well trained [9]. In this case, the patient would not have been treated on the first day due to the significant difference of target location and stomach volume. However, by using cutting-edge technology, all adaptive process was carried out while the patient was still on the couch. Respiratory gated radiotherapy with real-time deformable image registration-based beam control ensures prescribed radiation delivery to the target. PTV margin to compensate set-up error and organ motion can be greatly reduced for MRI-guided radiotherapy as compared to other image guidance planning and treatment system combinations.

For image guidance, it is well known that MR provides better contrast compared to CT, especially in gastrointestinal (GI) malignancies. CBCT has low discrimination power between soft tissues, which is critical when targeting GI tumors. As in this case, the stomach CT was unable to identify the exact tumor extent, whereas the image with MR offered a marked contrast of the tumor as shown. Additional radiation exposure is another issue. For standard mode CBCT, reported mean skin dose for pelvis imaging is 5.4 cGy per scan [10]. The radiation dose for CBCT can reach upto 1 Gy for the skin with daily imaging in conventional 30 fractions, which should be taken into account. Meanwhile, MR does not use ionizing radiation. This makes MR more appealing as a tool for daily image guidance. Considering high soft tissue contrast and non-exposure to ionizing radiation, MR guided radiotherapy may be a superior alternative to conventional on-board imaging or CBCT.

For this reported case, the role of salvage radiotherapy is pending due to limited a follow-up period of only six weeks. Although there was additional bleeding episode after treatment completion, the tumor was nearly resolved on post real-time endoscopic evaluation. Further studies with longer follow up utilizing a real-time MRI-guided system to make best of its ability of high precision treatment delivery to smaller volume and subsequently less treatment-related adverse events over previously available radiotherapy options are awaited.

## Conclusions

Despite short post-treatment follow-up limiting the efficacy review, this case report implies that MR guided radiotherapy can be a viable alternative for patients with stomach cancer who are unfit for both surgery and chemotherapy. With the help of real-time MR guidance and adaptive planning system, it is possible to safely deliver the radiation dose to focal lesions in the stomach. This experience may help to expand the role of radiotherapy as a salvage treatment in stomach cancer. However, further experience with larger cohort size and longer follow-up is warranted.
